# Assessing the Impact of Gepirone on Major Depressive Disorder ‐ A systematic review and meta analysis

**DOI:** 10.1002/alz.095197

**Published:** 2025-01-09

**Authors:** Mostafa A. Khalifa, Abdelmoneim Osama Salaheldin, Jana Ahmed Elsead, Fatama Azzam Aboud, Basmala Mohamed Korim, Shehab Sherif Abdalsttar, Abdalla Ahmed Mahmoud

**Affiliations:** ^1^ Faculty of Medicine, Cairo University, Cairo, Giza Egypt; ^2^ Faculty of Medicine, Cairo University, Cairo, giza Egypt; ^3^ Faculty of Medicine, Cairo University, Cairo Egypt; ^4^ College of Medicine, Arab Academy For Science, Technology and Maritime Transport, New Alamein, Matrouh Egypt

## Abstract

**Background:**

Major depression has been affecting more than 280 million of the general population, and this number continue to rise. Thus, impairing daily life activities. Research on Major depression has been since the 19th century for the sake of elevating symptoms with less side effects. The aim of our study is to assess the impact of a potential game changer in treatment of Major depression, Geprione

**Method:**

we have conducted a comprehensive search, including key databases such as Cochrane, PubMed, SCOPUS, and ScienceDirect, to ensure thorough coverage of all relevant literature, using appropriate key mesh terms. Search date ranged from 1987 to 2024. Randomized controlled studies published between that studied Patients with Major Depressive Disorder (MDD) diagnosed based on standardized criteria such as DSM‐5 with Geprione only as administration while comparing it to a placebo reflected as reduction in depressive symptoms for outcome were included. Exclusion criteria included populations with induced major depression such as substance abuse and breast‐feeding women, studies assessing other drugs from azapirones family than Geprione, with comparison to any drug other than placebo, and randomized controlled studies with incomplete outcome data. This systematic review was registered with PROSPERO [CRD42024510595] and followed PRISMA guidelines. Risk of bias was assessed using Cochrane library tool RoB 2 (revised tool for Risk of Bias in randomized trials). Data syntheses will be performed through Rev‐Man program

**Result:**

Seven Randomized controlled trails (n = 7) were included from 203 identified titles, after excluding duplicates (n = 13) and not fulfilling the inclusion and eligibility criteria (n = 183). A pilot study was conducted on 4 of these papers, revealing that all studies demonstrated that Gepirone is a potential treatment for major depression disorder and Our systematic review is currently in progress and is expected to be completed by the end of 2024

**Conclusion:**

The study views Gepirone as a potential treatment for major depression disorder with regard to fewer side effects and higher efficacy compared to standard treatment e.g., atypical antidepressants and tricyclic antidepressants. We encourage further research across variable populations for more robust evidence.

